# Aromatic Constituents from the Stems of *Astragalus membranaceus* (Fisch.) Bge. *var*. *Mongholicus* (Bge.) Hsiao

**DOI:** 10.3390/molecules21030354

**Published:** 2016-03-16

**Authors:** Jia Hao, Jian Li, Xiaoxia Li, Yanxia Liu, Jingya Ruan, Yongzhe Dong, Yi Zhang, Tao Wang

**Affiliations:** 1 Tianjin Key Laboratory of TCM Chemistry and Analysis, Institute of Traditional Chinese Medicine, Tianjin University of Traditional Chinese Medicine, 312 Anshan Road, Nankai District, Tianjin 300193, China; synthia2010@sina.com (J.H.); liuyanxia210@163.com (Y.L.); Ruanjy19930919@163.com (J.R.); dongyongzhe44@hotmail.com (Y.D.); 2 Tianjin State Key Laboratory of Modern Chinese Medicine, 312 Anshanxi Road, Nankai District, Tianjin 300193, China; beyondwill@126.com (J.L.); huifeidedouzi@yeah.net (X.L.)

**Keywords:** *Astragalus membranaceus* (Fisch.) Bge. *var*. *mongholicus* (Bge.) Hsiao., stem, structure elucidation, aromatic constituents, L6 cells, glucose consumption

## Abstract

Four new aromatic constituents, astraflavonoids A (**1**), B (**2**), C (**3**), and astramemoside A (**4**), along with sixteen known ones **5**–**20** were obtained from the stems of *A. membranaceus* (Fisch.) Bge. *var*. *mongholicus* (Bge.) Hsiao. Their structures were elucidated by chemical and spectroscopic methods. Among the known isolates, **14** was obtained from the Astragalus genus for the first time, while **7**–**12**, **18**–**20** were isolated from the species for the first time. The effects of the compounds obtained from the plant on glucose consumption were analyzed in differentiated L6 myotubes *in vitro*, whereby compounds **1**, **2**, **3**, **7**, **8**, **10**, **11**, **14**, **15** and **18** displayed significant promoting effects on glucose consumption in L6 myotubes. Among them, the activities of **1**, **2** and **7** were comparable to that of insulin, which suggested that these compounds may be involved in glucose metabolism and transport.

## 1. Introduction

*Astragalus membranaceus* (Fisch.) Bge. *var*. *mongholicus* (Bge.) Hsiao (AM), belongs to the Astragalus genus of the Leguminosae family. The main chemical constituents of the plant are flavonoids and terpenoids. During the course of our studies, 14 oleanane type saponins, including eight new ones, named astroolesaponins A, B, C_1_, C_2_, D, E_1_, E_2_, and F, have been obtained from its stems, and some of them showed depressing effects on triglyceride levels in sodium oleate-induced HepG2 cells [1]. During our continued research on this species, twenty aromatic constituents, including four new ones, were isolated from AM using SiO_2_ gel, ODS, Sephadex LH-20 column chromatography (CC) and preparative HPLC chromatography (Prep HPLC), and their structures were clearly determined by chemical and spectroscopic methods (^1^H-NMR, ^13^C-NMR, ^1^H-^1^H COSY, HSQC, HMBC, UV, IR, CD, and MS). Based on the evidences of previous activity reports on the Astragalus genus [1], the glucose consumption effects of the isolates were examined.

## 2. Results and Discussion

**Figure 1:**
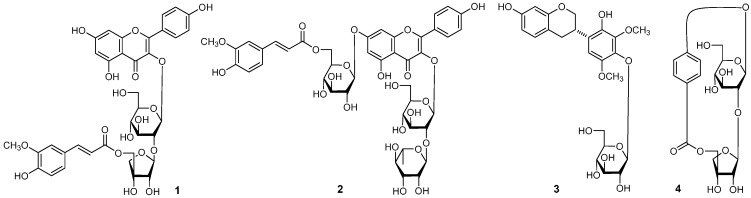
The new compounds **1**–**4** obtained from AM.

**Figure 2:**
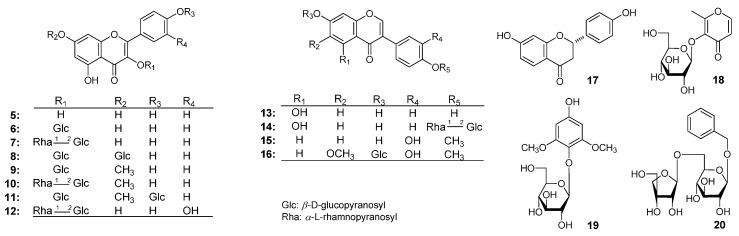
The known compounds **5**–**20** obtained from AM.

The 70% EtOH extract of AM was subjected to solvent partition, chromatographic isolation, and chemical and spectral analysis. As a result, four new compounds (Figure 1), astraflavonoids A–C (**1**–**3**) and astramemoside A (**4**), along with sixteen known ones (Figure 2), kaempferol (**5**) [2], kaempferol-3-*O*-β-d-glucoside (**6**) [3], kaempferol-3-*O*-(2-*O*-α-l-rhamnopyranosyl)-β-d-glucopyranoside (**7**) [4], kaempferol-3,7-di-*O*-β-d-glucopyranoside (**8**) [5], rhamnocitrin-3-*O*-β-d-glucopyranoside (**9**) [6], rhamnocitrin-3-*O*-neohesperoside (**10**) [7], complanatuside (**11**) [8], quercetin-3-*O*-β-d-neospheroside (**12**) [9], genistein (**13**) [10], sophorabioside (**14**) [11], calycosin (**15**) [12], odoratin-7-*O*-β-d-glucopyranoside (**16**) [13], (−)-liquiritigenin (**17**) [14,15], maltol-β-d-glucopyranoside (**18**) [16], 2,6-dimethoxy-4-hydroxyphenyl-1-*O*-β-d-glucopyranoside (**19**) [17], and benzyl-α-l-arabino-pyranosyl(1′′ → 6′)-β-d-glucopyranoside (**20**) [18] were obtained from it. Among the known isolates, compound **14** was isolated from the Astragalus genus for the first time, and **7**–**12**, **18**–**20** were isolated from the species for the first time. Herein, the isolation and identification of these compounds are described, as well as their effects on glucose consumption in L6 cells.

**Figure 3:**
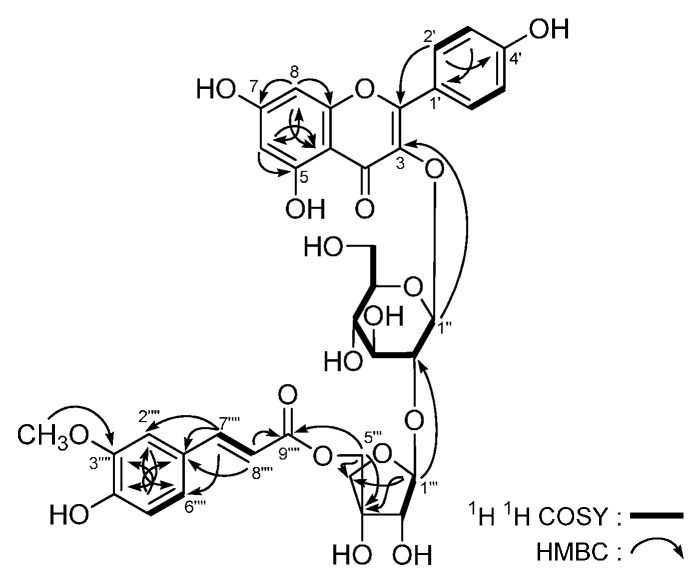
The main ^1^H-^1^H COSY and HMBC correlations of **1**.

Astraflavonoid A (**1**) was isolated as a yellow powder with ${\left[\alpha \right]}_{D}^{25}$ −69.8° (in MeOH). Its molecular formula is C_36_H_36_O_18_, as indicated by HR-Q-TOF-ESI-MS (*m*/*z* 755.1795 [M − H]^−^, calcd for C_36_H_35_O_18_, 755.1829). The IR spectrum showed absorption bands due to hydroxyl (3365 cm^−^^1^), α,β-unsaturated ester carbonyl (1693 cm^−^^1^), α,β-unsaturated ketone (1654 cm^−^^1^), aromatic ring (1604, 1512, 1453 cm^−^^1^), and ether functions (1071 cm^−^^1^). The ^1^H- and ^13^C-NMR (DMSO-*d*_6_, Table 1) spectra suggested the presence of the following moieties in **1**: kaempferol aglycon [δ 6.16 (1H, d, *J* = 1.5 Hz, H-6), 6.24 (1H, d, *J* = 1.5 Hz, H-8), 6.86 (2H, d, *J* = 9.0 Hz, H-3′,5′), 7.99 (2H, d, *J* = 9.0 Hz, H-2′,6′), 12.58 (1H, br. s, 5-OH)], *trans*-*p*-feruloyl [δ 3.81 (3H, s, 3′′′′-OCH_3_), 6.21 (1H, d, *J* = 16.0 Hz, H-8′′′′), 6.77 (1H, d, *J* = 8.0 Hz, H-5′′′′), 6.93 (1H, dd, *J* = 1.5, 8.0 Hz, H-6′′′′), 7.14 (1H, *J* = 1.5 Hz, H-2′′′′), 7.25 (1H, d, *J* = 16.0 Hz, H-7′′′′)], together with two anomeric proton signals [δ 5.40 (1H, br. s, H-1′′′), 5.66 (1H, d, *J* = 7.5 Hz, H-1′′)]. Moreover, the ^1^H-^1^H COSY experiment indicated the presence of partial structure indicated in bold lines (Figure 3). In the HMBC experiment, the long-range correlations from the following proton to carbon pairs were observed: δ_H_ 5.66 (H-1′′) to δ_C_ 132.7 (C-3); δ_H_ 5.40 (H-1′′′) to δ_C_ 75.9 (C-2′′); δ_H_ [4.15 (1H, d, *J* = 11.0 Hz), 4.27 (1H, d, *J* = 11.0 Hz), H2-5′′′] to δ_C_ 166.2 (C-9′′′′). Meanwhile, the ^13^C-NMR data for the sugar parts were in good agreement with those of isorhamnetin 3-*O*-(5-*O*-*trans*-feruloyl-β-d-apiofuranosyl)-(1 → 2)-β-d-glucopyranoside [δ 60.5 (C-6′′), 68.0 (C-5′′′), 70.2 (C-4′′), 73.8 (C-4′′′), 76.2 (C-2′′′), 75.7 (C-2′′), 77.0 (C-3′′), 77.4 (C-5′′), 77.6 (C-3′′′), 98.5 (C-1′′), 107.6 (C-1′′′)] [19]. Consequently, the structure of **1** was elucidated to be kaempferol 3-*O*-[(5-*O*-*trans*-*p*-feruloyl)-β-d-apiofuranosyl](1 → 2)-β-d-glucopyranoside.

**Figure 4:**
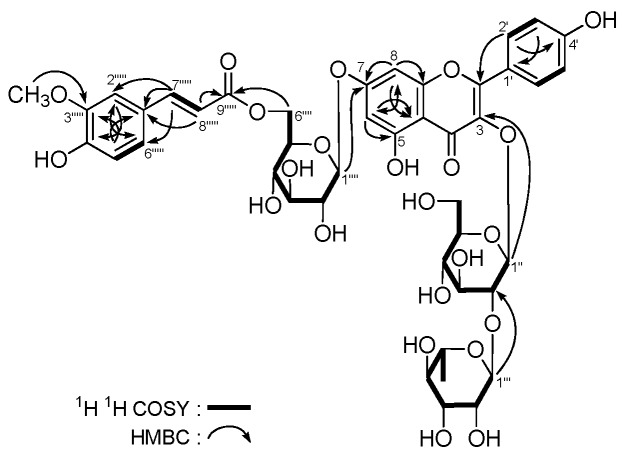
The main ^1^H-^1^H COSY and HMBC correlations of **2**.

Astraflavonoid B (**2**) displayed a negative optical rotation (${\left[\alpha \right]}_{D}^{25}$ −91.7° in MeOH). Its molecular formula, C_43_H_48_O_23_, was determined from the molecular ion peak at *m*/*z* 931.2525 [M − H]^−^ by HR-Q-TOF-ESI-MS measurement. Acid hydrolysis of **2** with 1 M HCl yielded d-glucose and l-rhamnose, which were identified on the basis of their retention times (HPLC) and optical rotations [20] The ^1^H-, ^13^C-NMR (DMSO-*d*_6_, Table 2) and various 2D NMR spectra indicated the presence of a kaempferol aglycon [δ 6.51 (1H, d, *J* = 1.5 Hz, H-6), 6.81 (1H, d, *J* = 1.5 Hz, H-8), 6.87 (2H, d, *J* = 9.0 Hz, H-3′,5′), 8.06 (2H, d, *J* = 9.0 Hz, H-2′,6′), 12.63 (1H, br. s, 5-OH)], *trans*-*p*-feruloyl [δ 3.80 (3H, s, 3′′′′′-OCH_3_), 7.28 (1H, *J* = 1.5 Hz, H-2′′′′′), 6.77 (1H, d, *J* = 8.5 Hz, H-5′′′′′), 7.04 (1H, dd, *J* = 1.5, 8.5 Hz, H-6′′′′′), 7.54 (1H, d, *J* = 16.0 Hz, H-7′′′′′), 6.46 (1H, d, *J* = 16.0 Hz, H-8′′′′′)], along with two β-d-glucopyranosyl [δ 5.18 (1H, d, *J* = 7.0 Hz, H-1′′′′), 5.67 (1H, d, *J* = 7.5 Hz, H-1′′)] and one α-l-rhamnopyranosyl [δ 0.79 (3H, d, *J* = 6.0 Hz, 6′′′-CH_3_), 5.10 (1H, br. s, H-1′′′)] moiety. Furthermore, according to the long-range correlations (Figure 4) observed from HMBC spectrum, the indicated locations of the *trans*-*p*-feruloyl, and sugar moieties in **2** could be determined.

**Figure 5:**
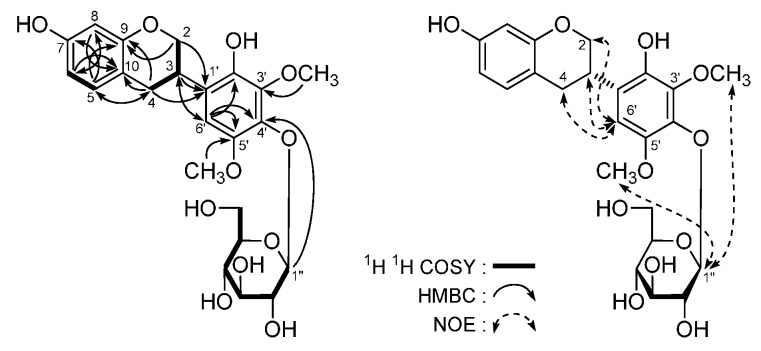
The main ^1^H-^1^H COSY, HMBC, and NOE correlations of **3**.

Astraflavonoid C (**3**) was obtained as a white powder with negative optical rotation (${\left[\alpha \right]}_{D}^{25}$ −26.7° in MeOH). Its molecular formula, C_23_H_28_O_11_, was established by HR-Q-TOF-ESI-MS (*m*/*z* 479.1578 [M − H]^−^; calcd for C_23_H_27_O_11_, 479.1559). Its acid hydrolysis yielded d-glucose [21]. The ^1^H-, ^13^C-NMR (CD_3_OD, Table 3) and DEPT spectra suggested the presence of one ABX-type aromatic ring, one pentasubstituted aromatic ring, one β-d-glucopyranosyl, and one oxygenated methylene, together with one methylene, one methane, and two methoxy groups. The ^1^H-^1^H COSY experiment indicated the presence of the partial structure indicated in bold lines (Figure 5). On the other hand, the long-range correlations from H_2_-2 to C-9, C-1′; H_2_-4 to C-5, C-9, C-10, C-1′; H-5 to C-4, C-7, C-9; H-6 to C-7, C-8, C-10; H-8 to C-6, C-7, C-10; H-6′ to C-3, C-2′, C-4′, C-5′; 3′-OCH_3_ to C-3′; 5′-OCH_3_ to C-5′; H-1′′ to C-4′ were clearly found in the HMBC spectra. Furthermore, the NOE correlations between δ_H_ 4.85 (H-1′′) and δ_H_ 3.83 (5′-OCH_3_), 3.90 (3′-OCH_3_) observed in the NOESY spectrum confirmed the substituted positions of two methoxy groups and the β-d-glucopyranosyl unit. Thus, the planar structure of **3** was identified as that of isoflavan glycoside. Finally, the CD spectrum of **3** displayed negative Cotton effect at 285 nm (Δε: −11.84), which indicated the absolute configuration of 3-position was *S* [20].

**Figure 6:**
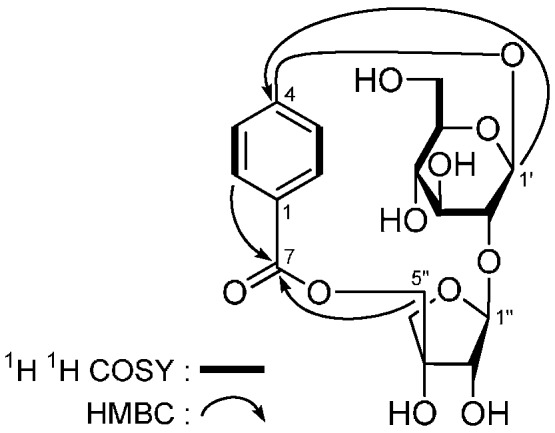
The main ^1^H-^1^H COSY and HMBC correlations of **4**.

Astramemoside A (**4**) was a white powder. Its molecular formula was deduced as C_18_H_22_O_11_ from the [M − H]^−^ quasi-molecular ion at *m*/*z* 413.1082 (calcd for C_18_H_21_O_11_, 413.1089) in the negative-ion HRESI-TOF-MS spectrum. The IR spectrum showed absorption bands due to hydroxyl (3373 cm^−1^), ester carbonyl (1721 cm^−^^1^), aromatic ring (1609, 1511, 1451 cm^−1^), and ether (1081 cm^−1^) functions. The ^1^H-, ^13^C-NMR (DMSO-*d*_6_, Table 4) and various 2D NMR spectra (Figure 6) suggested the presence of a *p*-hydroxybenzoic acid unit [δ_H_ 6.97 (2H, d, *J* = 9.0 Hz, H-3,5), 7.53 (2H, d, *J* = 9.0 Hz, H-2,6), δ_C_ 114.8 (C-3,5), 122.3 (C-1), 130.9 (C-2,6), 160.2 (C-4), 164.6 (C-7)].

Comparison of the ^1^H- and ^13^C-NMR spectroscopic data of **4** with those of **1** identified the sugar moiety to be β-d-apiofuranosyl(1 → 2)-β-d-glucopyranose. Moreover, the 5-position of the β-d-apiofuranosyl moiety was substituted too. Meanwhile, the long-range correlations from H-2,6 to C-4, C-7; H-1′ to C-4; H-1′′ to C-2′; H_2_-5′′ to C-7 observed in the HMBC experiment further proved the correctness of the above deductions.

**Figure 7:**
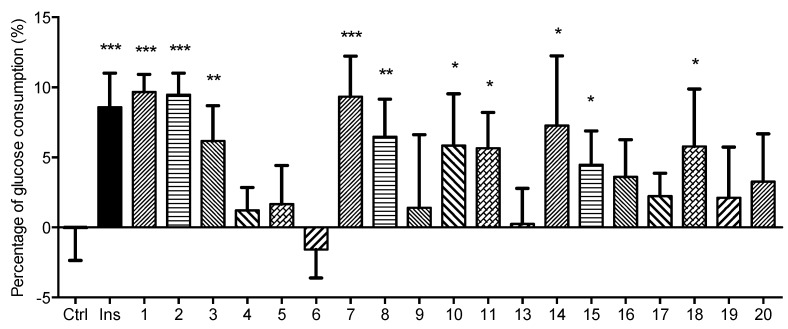
Effects of compounds **1**–**11** and **13**–**20** on glucose consumption in L6 myotubes. L6 myoblasts (1 × 10^4^ cells/well) were subcultured into 48-place multiwell plates in 2% FBS/DMEM for 7 days to form myotubes. The differentiated myotubes were kept in HBS with no serum or glucose for 2 h, and then were continue incubated in HBS containing 1 mg/mL d-glucose and 2% FBS with or without insulin (Ins, 2 μmol/L) or obtained compounds (30 μmol/L) for another 4 h. Then the glucose concentrations in the supernatant were detected using glucose assay kit and the percentage of glucose consumption in each well was calculated to express the results. Each value represents the mean ± S.E.M., *n* = 6. *** *p* < 0.001, ** *p* < 0.01, * *p* < 0.05 *vs.* control group (Ctrl).

The effects of the compounds obtained from AM on glucose consumption were analyzed in differentiated L6 myotubes *in vitro*. To create the assay method, standardization of basic parameters like differentiation time, the number of cells to seed, amount of d-glucose to be used and time of incubation were determined (data not shown). As shown in Figure 7, insulin increased the glucose consumption in L6 myotubes to about 4.76 ± 0.33 μg/well (*p* < 0.001) and the percentage of the raise reached about 9.01%, which serves as a positive control for our study. Among the tested compounds, **1**, **2**, **3**, **7**, **8**, **10**, **11**, **14**, **15** and **18** possessed significant promoting effects on glucose consumption in L6 myotubes, the glucose consumption of which were 5.02 ± 0.29, 4.92 ± 0.36, 3.20 ± 0.58, 4.86 ± 0.67, 3.37 ± 0.62, 3.04 ± 0.86, 2.94 ± 0.60, 3.96 ± 1.21, 2.44 ± 0.59 and 3.15 ± 1.00 μg/well, respectively. At the concentration of 30 μmol/L, compounds **1**, **2** and **7** led to 9.67%, 9.48% and 9.33% increments in glucose consumption, respectively, which was comparable to the effects of insulin. However, the other isolates showed no obvious effect on glucose consumption. These results indicated that some of the constituents in AM can stimulate glucose consumption in L6 myotubes to various degrees.

On the basis of the activity results of kaempferol and its glycosides, we can deduce that the glucosylation of 3-hydroxyl group would increase the effect on glucose consumption in L-6 cells, and disaccharide substitution at 3-hydroxyl showed a stronger activity than monosaccharide substitution. Due to the limited number of compounds, detailed studies are in progress to evaluate more kaempferol glycosides to clarify these structure-activity relationships.

## 3. Experimental Section

### 3.1. General Information

The following instruments were used to obtain physical data: optical rotations were recorded on an Autopol IV automatic polarimeter (*l* = 50 mm; Rudolph Research Analytical, Hackettstown, NJ, USA). IR and UV spectra were determined on 640-IR FT-IR (Varian Australia Pty Ltd., Mulgrave, Australia) and Cary 50 UV-Vis spectrophotometers, respectively (Varian, Inc., Hubbardsdon, MA, USA). ^1^H- and ^13^C-NMR spectra were measured on a Bruker 500 MHz NMR spectrometer at 500 MHz for ^1^H- and 125 MHz for ^13^C-NMR (Bruker BioSpin AG Industriestrasse 26 CH-8117, Fällanden, Switzerland). Negative-ion HR-ESI-Q-TOF-MS were recorded on an Aglient 6520 Q-TOF mass spectrometer (Agilent Corp., Santa Clara, CA, USA).

The following supports were used for chromatography: a macroporous synthetic resin (D101) (Haiguang Chemical Co., Ltd., Tianjin, China), SiO_2_ gel (74–149 μm, Qingdao Haiyang Chemical Co., Ltd., Qingdao, China), and ODS (50 μm, YMC Co., Ltd., Tokyo, Japan). Prep HPLC was performed on an ODS column (Cosmosil 5C18-MS-II, Tokyo, Japan; Φ = 20 mm, *l* = 250 mm, flow rate 9.0 mL/min), and the eluate was monitored with a UV detector (Shimadzu RID-10ª UV-vis, Shimadzu Co. Ltd., Kyoto, Japan).

### 3.2. Plant Material

The stems of *Astragalus membranaceus* (Fisch.) Bge. var. *mongholicus* (Bge.) Hsiao. were collected from Gansu Province, China, and identified by Dr. Li Tianxiang (Experiment Teaching Department, Tianjin University of Traditional Chinese Medicine, Tianjin, China). The voucher specimen was deposited at the Academy of Traditional Chinese Medicine of Tianjin University of TCM.

### 3.3. Extraction and Isolation

The stems of AM were dealt with by the method described before [1] to give the 95% EtOH eluate from D101 macroporous resin CC. The 95% EtOH eluate (90.6 g) was subjected to SiO_2_ gel CC to yield fourteen fractions (Fr. 1–14). Fraction 2 (0.16 g) was purified by Prep HPLC [MeOH–H_2_O (55:45, *v*/*v*)] to afford calycosin (**15**, 13.2 mg). Using the same isolation conditions as fraction 2, kaempferol (**5**, 8.7 mg), genistein (**13**, 7.2 mg), and (−)-liquiritigenin (**17**, 8.8 mg) were obtained from fraction 3 (0.78 g). Fraction 6 (7.0 g) was isolated by ODS CC, and purified by Prep HPLC to yield astraflavonoid C (**3**, 25.0 mg), kaempferol-3-*O*-β-d-glucoside (**6**, 43.8 mg), rhamnocitrin-3-*O*-β-d-glucopyranoside (**9**, 52.5 mg), and odoratin-7-*O*-β-d-glucopyranoside (**16**, 14.4 mg). Fraction 7 (6.9 g) was subjected to SiO_2_ gel, ODS, Sephadex LH-20 CC and Prep HPLC to furnish astraflavonoid A (**1**, 8.5 mg), maltol-β-d-glucopyranoside (**18**, 59.0 mg), and 2,6-dimethoxy-4-hydroxyphenyl-1-*O*-β-d-glucopyranoside (**19**, 12.2 mg). Fraction 8 (7.4 g) was subjected to ODS and Sephadex LH-20 CC, along with Prep HPLC to afford benzyl-α-l-arabinopyranosyl(1′′ → 6′)-β-d-glucopyranoside (**20**, 46.4 mg). Fraction 9 (7.0 g) was subjected to ODS CC, and purified by Prep HPLC to give rhamnocitrin-3-*O*-neohesperoside (**10**, 46.3 mg) and sophorabioside (**14**, 38.1 mg). Fraction 10 (12.0 g) was isolated by Sephadex LH-20 CC and Prep HPLC to yield complanatuside (**11**, 12.4 mg). Fraction 12 (8.2 g) was separated by Prep HPLC to provide astraflavonoid B (**2**, 22.2 mg), astramemoside A (**4**, 6.7 mg), kaempferol-3-*O*-(2-*O*-α-l-rhamnopyranosyl)-β-d-glucopyranoside (**7**, 14.5. mg) and kaempferol-3,7-di-*O*-β-d-glucopyranoside (**8**, 11.2 mg). Fraction 13 (12.1 g) was subjected to Sephadex LH-20 CC and further purified by Prep HPLC to afford quercetin-3-*O*-β-d-neospheroside (**12**, 2.2 mg).

### 3.4. Compound Characterization

*Astraflavonoid A* (**1**): Yellow powder; ${\left[\alpha \right]}_{D}^{25}$ −69.8° (*c* = 0.61, MeOH); UV (MeOH) λ_max_ (log ε) 268 (3.99), 292 (3.94, sh), 329 (4.06); IR (KBr) ν_max_ 3365, 2926, 2855, 1693, 1654, 1604, 1512, 1453, 1361, 1275, 1178, 1123, 1071, 1026, 839 cm^−1^; ^1^H- and ^13^C-NMR (DMSO-*d*_6_) data see Table 1; Negative-ion mode HR-Q-TOF-ESI-MS *m*/*z* 755.1795 [M − H]^−^ (calcd for C_36_H_35_O_18_, 755.1829).

*Astraflavonoid B* (**2**): Yellow powder; ${\left[\alpha \right]}_{D}^{25}$ −91.7° (*c* = 0.87, MeOH); UV (MeOH) λ_max_ (log ε) 267 (4.19), 292 (4.08, sh), 325 (4.20); IR (KBr) ν_max_ 3365, 2926, 1698, 1652, 1599, 1512, 1453, 1346, 1275, 1210, 1179, 1124, 1071, 840 cm^−1^; ^1^H- and ^13^C-NMR (DMSO-*d*_6_) data see Table 2; Negative-ion mode HR-Q-TOF-ESI-MS *m*/*z* 931.2525 [M − H]^−^ (calcd for C_43_H_47_O_23_, 931.2514).

*Astraflavonoid C* (**3**): White powder; ${\left[\alpha \right]}_{D}^{25}$ −26.7° (*c* = 0.89, MeOH); CD (*c* = 0.0021 M, MeOH) Δε (λnm) − 11.84 (285); UV (MeOH) λ_max_ (log ε) 228 (4.07, sh), 284 (3.68); IR (KBr) ν_max_ 3367, 2938, 2848, 1622, 1595, 1508, 1461, 1367, 1156, 1109, 1071, 1024, 847 cm^−1^; ^1^H- and ^13^C-NMR (CD_3_OD) data see Table 3; Negative-ion mode HR-Q-TOF-ESI-MS *m*/*z* 479.1578 [M − H]^−^ (calcd for C_23_H_27_O_11_, 479.1559).

*Astramemoside A* (**4**): White powder; ${\left[\alpha \right]}_{D}^{25}$ −21.0° (*c* = 0.39, MeOH); UV (MeOH) λ_max_ (log ε) 249 (3.41); IR (KBr) ν_max_ 3373, 2924, 2889, 1721, 1609, 1511, 1451, 1401, 1374, 1270, 1242, 1184, 1081, 1003, 848 cm^−1^; ^1^H- and ^13^C-NMR (DMSO-*d*_6_) data see Table 4; Negative-ion mode HR-Q-TOF-ESI-MS *m*/*z* 413.1082 [M − H]^−^ (calcd for C_18_H_21_O_11_, 413.1089).

### 3.5. Acid Hydrolysis of **2** and **3**

A solution of compounds **2** and **3** (each 1.5 mg) was treated with HCl using the same method and conditions as reported in [21]. Identification of l-rhamnose (i) from **2**; and d-glucose (ii) from **2** and **3** by comparison of its retention time and optical rotation with those of authentic sample, *t*_R_: (i) 7.5 min (l-rhamnose, negative optical rotation); and (ii) 14.1 min (d-glucose, positive optical rotation).

### 3.6. Glucose Consumption Assay of Compounds Obtained from AM

#### 3.6.1. Materials

L6 rat skeletal myoblasts cell line (Cell Resource Center, IBMS, CAMS/PUMC, Beijing, China), Dulbecco’s modified Eagle’s medium (DMEM, Thermo Scientific, Logan, UT, USA), fetal bovine serum (FBS, Thermo Scientific), streptomycin and penicillin G (Thermo Scientific), glucose assay kit (BioSino Bio-technology and Science Inc., Beijing, China), insulin (Sigma, St. Louis, MO, USA), d-glucose (Solarbio Bio-technology and Science Inc., Beijing, China China).

#### 3.6.2. Cell Culture and Differentiation

Stock cultures of L6 myoblasts were grown in 10% (*v*/*v*) FBS/DMEM supplemented with streptomycin (100 μg/mL) and penicillin G (100 U/mL) at 37°C under 5% CO_2_ atmosphere and maintained below 70% confluence. For differentiation into L6 myotubes, cells were cultured at a density of 1 × 10^4^ cells/well on 48-place multiwell plates (Costar, Washington, DC, USA) and the medium was switched to 2% (*v*/*v*) FBS/DMEM for 7 days, with medium changes every second day prior to use in our experiments.

#### 3.6.3. Determination of Glucose Consumption in L6 Myotubes

This method was based on some literatures [22,23,24] with slight modifications. Briefly, the 7-day-old myotubes were serum and glucose-deprived kept in HEPES-buffered saline (HBS, 20 mmol/L HEPES, 2 mmol/L sodium pyruvate, 136 mmol/L NaCl, 2.7 mmol/L KCl, 2 mmol/L KH_2_PO_4_, 10 mmol/L Na_2_HPO_4_, 2.5 mmol/L MgSO_4_, and 1 mmol/L CaCl_2_, pH 7.4) for 2 h, and were thereafter incubated in HBS containing 1 mg/mL d-glucose and 2% FBS with or without insulin (Ins, 2 μmol/L) or obtained compounds (**1**–**11**, **13**–**20**, 30 μmol/L each) for another 4 h. Then glucose concentrations in the supernatant were determined using glucose assay kit (GOD-POD colorimetric method) and the ratio of glucose consumption in each well was calculated for further comparison. Results were expressed as a percentage of glucose consumption:

(1) Percentage of glucose consumption (%) = [(glucose surplus of control − glucose surplus of sample)/glucose surplus of control] × 100%

## 4. Conclusions

In summary, four new aromatic constituents, astraflavonoids A–C (**1**–**3**), and astramemoside A (**4**), along with sixteen known ones **5**–**20** were obtained from the 70% EtOH extract of AM. Among the known isolates, **14** was isolated from the Astragalus genus for the first time, and compounds **7**–**12**, **18**–**20** were isolated from the species for the first time. Their structures were elucidated by chemical and spectroscopic methods. The effects of the compounds obtained from AM on glucose consumption were analyzed in differentiated L6 myotubes *in vitro*. As results, compounds **1**, **2**, **3**, **7**, **8**, **10**, **11**, **14**, **15** and **18** possessed significant promotion effects on glucose consumption in L6 myotubes. Among them, the activities of **1**, **2** and **7** were comparable to that of insulin, which suggested that these AM compounds may be involved in glucose metabolism and transportat. On the basis of the activity results, the structure-activity was discussed. Glucose consumption plays a role in cellular energy homeostasis. This process includes glucose uptake, translocation, glucose storage, involves many key kinase, including AMP-activated protein kinase, phosphoinositide 3-kinase, glycogen synthase kinase, and so on. Further studies will be carried out to elucidate the mechanism of action of these and other kaempferol derivatives on glucose consumption.

## Figures and Tables

**Table 1:** ^1^H- and ^13^C-NMR data for **1** in DMSO-*d*_6_.

No.	δ_C_	δ_H_ (*J* in Hz)	No.	δ_C_	δ_H_ (*J* in Hz)
2	155.4	-	6′′	60.6	3.29 (m, overlapped)
3	132.7	-			3.55 (br. d, *ca*. 12)
4	177.1	-	1′′′	107.7	5.40 (br. s)
5	161.2	-	2′′′	76.3	3.75 (br. s)
6	98.5	6.16 (d, 1.5)	3′′′	77.5	-
7	163.9	-	4′′′	73.6	3.57 (d, 9.5)
8	93.3	6.24 (d, 1.5)			3.95 (d, 9.5)
9	156.0	-	5′′′	67.6	4.15 (d, 11.0)
10	103.9	-			4.27 (d, 11.0)
1′	120.9	-	1′′′′	125.4	-
2′,6′	130.6	7.99 (d, 9.0)	2′′′′	110.7	7.14 (d, 1.5)
3′,5′	114.9	6.86 (d, 9.0)	3′′′′	147.7	-
4′	159.8	-	4′′′′	149.1	-
5-OH	-	12.58 (br. s)	5′′′′	115.3	6.77 (d, 8.0)
1′′	98.3	5.66 (d, 7.5)	6′′′′	122.9	6.93 (dd, 1.5, 8.0)
2′′	75.9	3.49 (dd, 7.5, 9.0)	7′′′′	144.5	7.25 (d, 16.0)
3′′	76.9	3.42 (dd, 9.0, 9.0)	8′′′′	114.0	6.21 (d, 16.0)
4′′	70.2	3.10 (dd, 9.0, 9.0)	9′′′′	166.2	-
5′′	77.4	3.09 (m)	3′′′′-OCH_3_	55.5	3.81 (s)

**Table 2:** ^1^H- and ^13^C-NMR data for **2** in DMSO-*d*_6_.

No.	δ_C_	δ_H_ (*J* in Hz)	No.	δ_C_	δ_H_ (*J* in Hz)
2	155.8	-	2′′′	70.5	3.75 (br. s)
3	132.9	-	3′′′	70.4	3.46 (dd, 3.0, 9.0)
4	177.4	-	4′′′	71.8	3.14 (dd, 9.0, 9.0)
5	160.9	-	5′′′	68.2	3.72 (m)
6	99.6	6.51 (d, 1.5)	′′′	17.2	0.79 (d, 6.0)
7	162.6	-	1′′′′	99.1	5.18 (d, 7.0)
8	94.4	6.81 (d, 1.5)	2′′′′	73.0	3.32 (dd, 7.0, 9.0)
9	156.6	-	3′′′′	76.1	3.36 (dd, 9.0, 9.0)
10	105.6	-	4′′′′	69.3	3.31 (m, overlapped)
1′	120.6	-	5′′′′	73.8	3.80 (m)
2′,6′	130.7	8.06 (d, 9.0)	6′′′′	63.0	4.20 (dd, 5.5, 12.0)
3′,5′	115.0	6.87 (d, 9.0)			4.44 (br. d, *ca*. 12)
4′	160.0	-	1′′′′′	125.4	-
5-OH	-	12.63 (br. s)	2′′′′′	110.9	7.28 (d, 1.5)
1′′	98.2	5.67 (d, 7.5)	3′′′′′	147.8	-
2′′	77.5	3.46 (dd, 7.5, 9.0)	4′′′′′	149.3	-
3′′	77.2	3.40 (dd, 9.0, 9.0)	5′′′′′	115.4	6.77 (d, 8.5)
4′′	70.1	3.09 (dd, 9.0, 9.0)	6′′′′′	123.1	7.04 (dd, 1.5, 8.5)
5′′	77.4	3.09 (m)	7′′′′′	145.2	7.54 (d, 16.0)
6′′	60.7	3.28 (dd, 4.5, 11.5)	8′′′′′	114.0	6.46 (d, 16.0)
		3.56 (br. d, *ca*. 12)	9′′′′′	166.5	-
1′′′	100.5	5.10 (br. s)	3′′′′′-OCH_3_	55.6	3.80 (s)

**Table 3:** ^1^H- and ^13^C-NMR data for **3** in CD_3_OD.

No.	δ_C_	δ_H_ (*J* in Hz)	No.	δ_C_	δ_H_ (*J* in Hz)
2	71.6	3.84 (dd, 8.5, 9.0)	4′	141.3	-
		4.33 (dd, 2.0, 9.0)	5′	141.6	-
3	32.4	3.80 (m)	6′	109.8	6.42 (s)
4	32.5	2.75 (m)	1′′	105.6	4.85 (d, 9.0)
5	131.3	6.85 (d, 8.0)	2′′	75.6	3.43 (m, overlapped)
6	109.2	6.32 (dd, 2.5, 8.0)	3′′	77.9	3.43 (m, overlapped)
7	157.6	-	4′′	71.5	3.36 (dd, 6.5, 9.5)
8	103.9	6.25 (d, 2.5)	5′′	78.2	3.24 (m)
9	156.3	-	6′′	62.8	3.70 (dd, 5.0, 11.5)
10	114.9	-			3.85 (dd, 2.0, 11.5)
1′	133.1	-	3′-OCH_3_	61.9	3.90 (s)
2′	148.8	-	5′-OCH_3_	61.3	3.83 (s)
3′	147.6	-			

**Table 4:** ^1^H- and ^13^C-NMR data for **4** in DMSO-*d*_6_.

No.	δ_C_	δ_H_ (*J* in Hz)	No.	δ_C_	δ_H_ (*J* in Hz)
1	122.3	-	6′	60.2	3.45 (dd, 5.5, 12.0)
2,6	130.9	7.53 (d, 9.0)			3.65 (br. d, *ca*. 12)
3,5	114.8	6.97 (d, 9.0)	1′′	107.7	5.34 (br. s)
4	160.2	-	2′′	76.3	3.65 (br. s)
7	164.6	-	3′′	77.5	-
1′	96.8	5.18 (d, 7.0)	4′′	73.4	3.80 (d, 9.5)
2′	75.3	3.53 (dd, 7.0, 9.0)			4.31 (d, 9.5)
3′	76.7	3.52 (dd, 9.0, 9.0)	5′′	68.0	3.87 (d, 11.0)
4′	69.5	3.20 (dd, 9.0, 9.0)			4.32 (d, 11.0)
5′	76.7	3.52 (m)			
